# Supplementary figures and images for: An Age Effect of Rumen Microbiome in Dairy Buffaloes Revealed by Metagenomics
Source: Microorganisms. 2022 Jul 25;10(8):1491. doi: 10.3390/microorganisms10081491 (PMC9332492; doi:10.3390/microorganisms10081491)

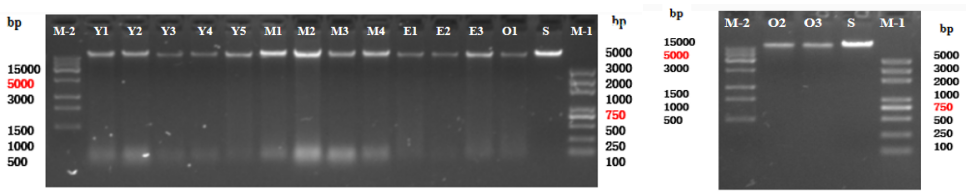

Supplement: Supplementary file 1 [file microorganisms-10-01491-s001.zip › Figure S1. Gel images for the DNA integrity and purity..png]

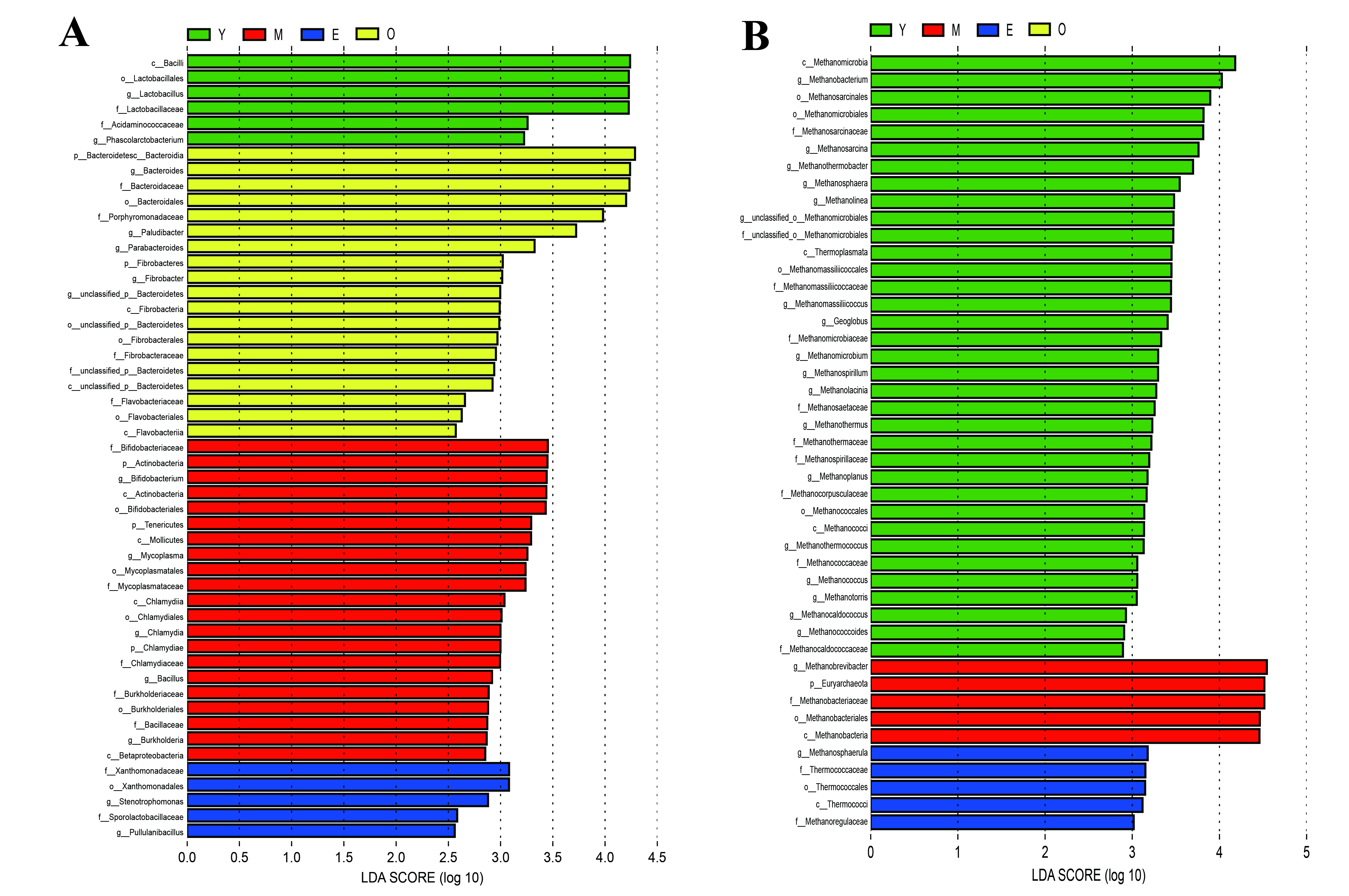

Supplement: Supplementary file 1 [file microorganisms-10-01491-s001.zip › Figure S2. Histogram of the LDA scores computed for differentially abundant rumen (bacteria & archaea).jpg]

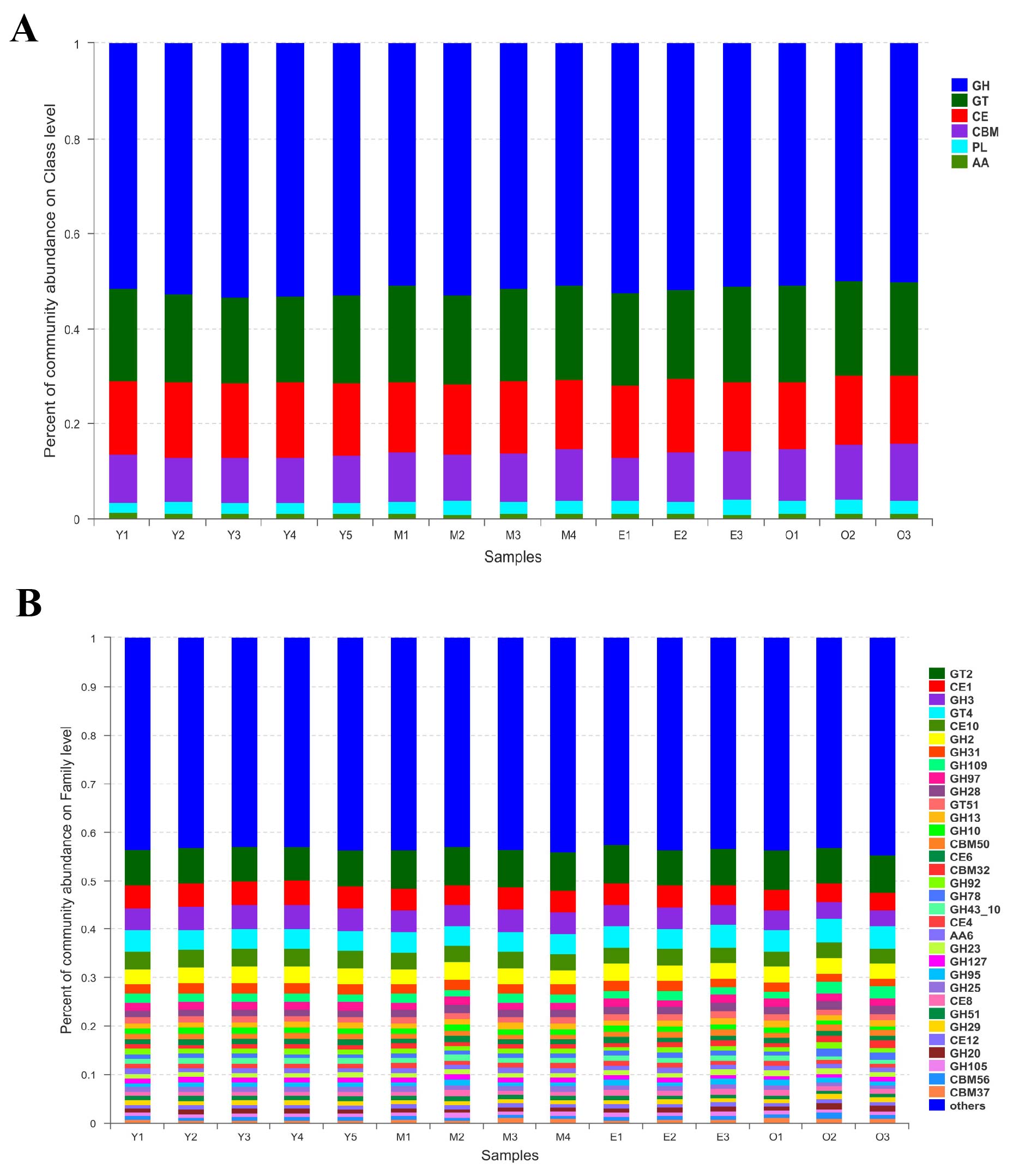

Supplement: Supplementary file 1 [file microorganisms-10-01491-s001.zip › Figure S3. Composition of predicated CAZyme genes of dairy buffalo rumen microbiota-01.jpg]

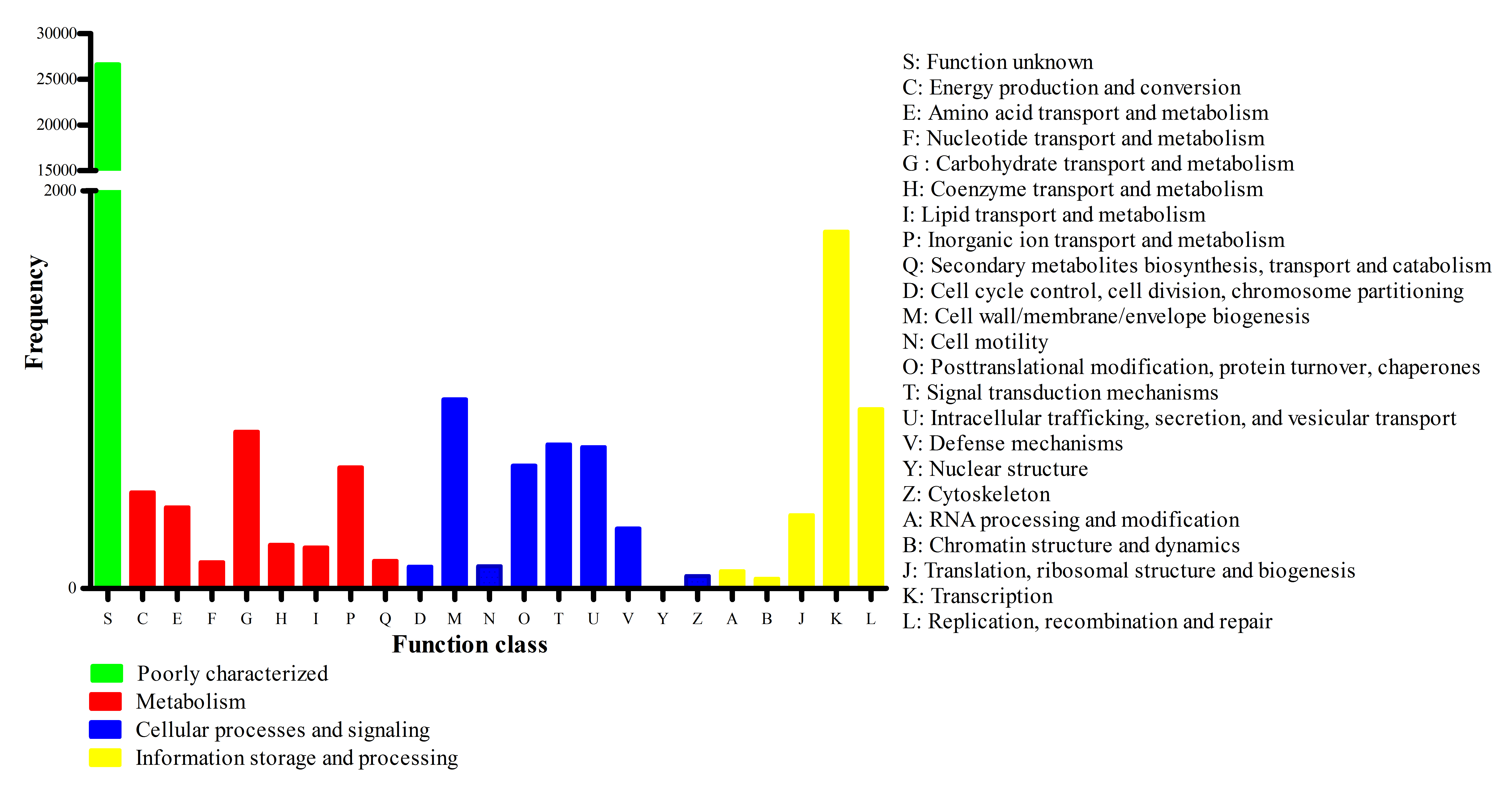

Supplement: Supplementary file 1 [file microorganisms-10-01491-s001.zip › Figure S4. Distribution of COG functional annotation of identified genes from dairy buffalo rumen microbiota..jpg]
